# Persistent Increase in Serum Ferritin Levels despite Converting to Permanent Vascular Access in Pediatric Hemodialysis Patients: Pediatric Nephrology Research Consortium Study

**DOI:** 10.3390/jcm12134251

**Published:** 2023-06-25

**Authors:** Ali Mirza Onder, Md Abu Yusuf Ansari, Fang Deng, Matthew M. Grinsell, Larry Patterson, Jennifer Jetton, Sahar Fathallah-Shaykh, Daniel Ranch, Diego Aviles, Lawrence Copelovitch, Eileen Ellis, Vimal Chadha, Ayah Elmaghrabi, Jen-Jar Lin, Lavjay Butani, Maha Haddad, Olivera Marsenic, Paul Brakeman, Raymond Quigley, H. Stella Shin, Rouba Garro, Rupesh Raina, Craig B. Langman

**Affiliations:** 1 Division of Pediatric Nephrology, Batson Children’s Hospital of Mississippi, University of Mississippi, Jackson, MS 39216, USA; 2 Division of Pediatric Nephrology, Nemours Children’s Hospital, Delaware, Wilmington, DE 19803, USA; 3 Department of Data Science, University of Mississippi Medical Center, Jackson, MS 39216, USA; mansari@umc.edu; 4 Kidney Diseases Division, Feinberg School of Medicine, Northwestern University, Ann and Robert H Lurie Children’s Hospital of Chicago, Chicago, IL 60611, USA; dengfang1997@126.com (F.D.); c-langman@northwestern.edu (C.B.L.); 5 Division of Pediatric Nephrology, Primary Children’s Hospital, University of Utah, Salt Lake City, UT 84112, USA; matt.grinsell@hsc.utah.edu; 6 Division of Pediatric Nephrology, Children’s National Health System, Washington, DC 20010, USA; lpatterson@childrensnational.org; 7 Division of Nephrology, Dialysis and Transplantation, University of Iowa Stead Family Children’s Hospital, Iowa City, IA 52242, USA; jennifer-jetton@uiowa.edu; 8 Division of Pediatric Nephrology, Children’s of Alabama, University of Alabama, Birmingham, AL 35233, USA; sfathallah@peds.uab.edu; 9 Division of Pediatric Nephrology, University of Texas Health Science Center, San Antonio, TX 78229, USA; ranch@uthscca.edu; 10 Division of Pediatric Nephrology, Children’s Hospital New Orleans, LSU Heath School of Medicine, New Orleans, LA 70118, USA; davile@lsuhsc.edu; 11 Division of Nephrology, Children’s Hospital of Philadelphia, Philadelphia, PA 19104, USA; copelovitch@email.chop.edu; 12 Division of Pediatric Nephrology, Arkansas Children’s Hospital, Little Rock, AR 72202, USA; elliseileenn@uams.edu; 13 Division of Pediatric Nephrology, Children’s Mercy Hospital, Kansas City, MO 64108, USA; vchadha@cmh.edu; 14 Division of Pediatric Nephrology, Children’s Medical Center Dallas, UT Southwestern, Dallas, TX 75235, USA; aelmaghrabi@akdhc.com (A.E.); raymond.quigley@utsouthwestern.edu (R.Q.); 15 Division of Pediatric Nephrology, Brenner Children’s Hospital, Wake Forest University, Winston Salem, NC 27157, USA; jelin@wakehealth.edu; 16 Division of Pediatric Nephrology, UC Davis Children’s Hospital, Sacramento, CA 95817, USA; lbutani@ucdavis.edu (L.B.); mnhaddad@ucdavis.edu (M.H.); 17 Division of Pediatric Nephrology, Lucile Packard Children’s Hospital, Stanford University School of Medicine, Stanford, CA 94305, USA; oljamc@stanford.edu; 18 Division of Pediatric Nephrology, UCSF Benioff Children’s Hospital, San Francisco, CA 94158, USA; paul.brakeman@ucsf.edu; 19 Division of Pediatric Nephrology, Children’s Healthcare of Atlanta, Atlanta, GA 30322, USA; stella.shin@emory.edu (H.S.S.); rouba.garro@emory.edu (R.G.); 20 Division of Pediatric Nephrology, Akron Children’s Hospital, Akron, OH 44308, USA; rraina@akronchildrens.org

**Keywords:** arteriovenous fistula, arteriovenous graft, ferritin, pediatric, hemodialysis

## Abstract

Our objective was to examine serum ferritin trends after conversion to permanent vascular access (PVA) among children who started hemodialysis (HD) using tunneled cuffed catheters (TCC). Retrospective chart reviews were completed on 98 subjects from 20 pediatric HD centers. Serum ferritin levels were collected at the creation of PVA and for two years thereafter. There were 11 (11%) arteriovenous grafts (AVG) and 87 (89%) arteriovenous fistulae (AVF). Their mean TCC use was 10.4 ± 17.3 months. Serum ferritin at PVA creation was elevated at 562.64 ± 492.34 ng/mL, increased to 753.84 ± 561.54 ng/mL (*p* = < 0.001) in the first year and remained at 759.60 ± 528.11 ng/mL in the second year (*p* = 0.004). The serum ferritin levels did not show a statistically significant linear association with respective serum hematocrit values. In a multiple linear regression model, there were three predictors of serum ferritin during the first year of follow-up: steroid-resistant nephrotic syndrome as primary etiology (*p* = 0.035), being from a center that enrolled >10 cases (*p* = 0.049) and baseline serum ferritin level (*p* = 0.017). Increasing serum ferritin after conversion to PVA is concerning. This increase is not associated with serum hematocrit trends. Future studies should investigate the correlation of serum transferrin saturation and ferritin levels in pediatric HD patients.

## 1. Introduction

Optimal anemia management is essential for children with end-stage renal disease (ESRD) to reach target growth, optimize cognitive development and maintain good cardiovascular health [1,2,3]. With the discovery of erythropoiesis-stimulating agents (ESA), such as recombinant human erythropoietin and the improved safety profile of parenteral iron formulations, there has been significant improvement in anemia treatment options for these children [4,5]. These advances significantly decreased the need for blood transfusions [2,6]. Less exposure to blood cells not only decreased the risk of sensitization against the HLA antigenic epitopes but also eliminated the concerns for dialysis-associated hemochromatosis and dangerously elevated iron overloads in these patients [7,8,9]. The National Kidney Foundation’s Kidney Disease Outcomes Quality Initiative (KDOQI) Anemia Treatment Guidelines recommends keeping the serum transferrin saturation >20% using parenteral iron supplementation for the best efficacy of ESA use [6]. Serum ferritin is utilized as the surrogate marker of body iron stores, and it is recommended that parenteral iron should not be administered if serum ferritin level is >800 ng/mL or serum transferrin saturation is >50% [6,10,11,12].

Serum ferritin has evolved to be a marker of body iron stores and is utilized to regulate parenteral iron therapy in ESRD patients [12,13]. Tissue ferritin is the main storage molecule for iron, where iron is stored in soluble form and protected from oxidation and is an important regulator for the release of iron [14,15]. The function of serum ferritin, on the other hand, is not clearly demonstrated. Serum ferritin does not contain Fe molecules, is not responsible for iron transport and is speculated to reach circulation due to the leakage of tissue ferritin [14,15]. Low levels of serum ferritin are well associated with depleted iron stores [15,16]. However, high serum ferritin may not automatically translate to increased body iron stores, as ferritin is a well-established acute-phase reactant and high levels may be secondary to ongoing inflammation that may be dialysis-associated or even dialysis-unrelated [17,18]. In the light of new evidence, 2012 KDIGO anemia guidelines recommended higher cut-off values of serum transferrin saturation (<30%) and accepted higher serum ferritin levels (<500 ng/mL) for the initiation of parenteral iron treatments [19].

Both ESRD and hemodialysis (HD) are thought of as inflammatory conditions. The type of vascular access used may contribute to the baseline level of inflammation. It was shown that non-infected tunneled cuffed catheters (TCC) are associated with increased inflammatory markers compared to arteriovenous fistulae (AVF) [20]. The elevated pro-inflammatory markers in children with ESRD on renal replacement treatment respond to aspirin therapy [21]. Interestingly, high serum ferritin levels were associated with elevated markers of inflammation and malnutrition for adult HD population [22,23]. Most concerning, elevated serum ferritin was also reported as a predictor of mortality for adult ESRD patients [24,25,26].

With this background of anemia management for pediatric HD patients and the dual purpose of serum ferritin, it is important to study the impact of conversion from TCC to AVF/AVG on serum ferritin levels for children undergoing chronic HD [27,28]. Our objective for this study was to observe the effect of switching from TCC to AVF/arteriovenous graft (AVG) on serum ferritin levels over two years of follow-up [29,30]. This multicenter, contemporary pediatric HD cohort, minimizing the biases that result from single-center studies, was used to evaluate the impact of this switch [29,30]. We attempted to examine serum ferritin level trajectories in relation to other HD efficacy markers, such as single pool Kt/V, serum albumin and, specifically, serum hematocrit levels [30].

## 2. Methods

The basic structure and methodology of this study have been described in the previous reports that were published using the same database [28,30]. In summary, this is a multi-center, retrospective chart review analysis. Institutional Review Board (IRB) approval for data collection from patients’ charts was obtained by each participating center. Twenty pediatric dialysis centers from Pediatric Nephrology Research Consortium (PNRC) conducted chart reviews for retrospective data collection. Inclusion criteria included first AVF/AVG creation between 1 January 2009 and 31 December 2013. All subjects were younger than 19 years of age at their first AVF/AVG creation, all had been on HD for at least three months and all were using TCC as their vascular access prior to the switch to AVF/AVG. The outcome variable investigated for this study was serum ferritin levels. Single-pool Kt/V, serum albumin and serum hematocrit (Hct) were also investigated in relation to serum ferritin outcomes. The above-mentioned variables were collected at the time of AVF/AVG creation and then in the first year and second year of AVF/AVG. On an important note, all the laboratory tests were collected when the subjects were otherwise at their baseline state. None of these results were obtained when a subject was admitted to the hospital or was receiving treatment for catheter-related bacteremia. This was emphasized and clarified to all the contributing centers during the data collection process. Various demographic, clinical and laboratory data elements were further collected for data modeling purposes. Please refer to previous publications for a detailed explanation of these variables [28,30]. 

The primary outcome was serum ferritin level in the first year and second year of AVF/AVG. Serum ferritin level at the time of AVF/AVG creation were taken as the TCC-associated level. The mean TCC vintage at AVF/AVG creation was almost 1 year (10.4 ± 17.3 months). Monthly serum ferritin levels other than primary outcome values were not included in the analysis. The secondary outcomes were serum Hct, serum albumin and single pool Kt/V in the first year and second year of PVA. These children’s parenteral iron-dosing protocols, monthly erythropoietin dose exposure or daily protein intake and anthropometric measurements were not included in the dataset. 

Serum ferritin trajectories were evaluated in two groups, according to the change in ferritin levels. The “unchanged ferritin group” included those subjects whose ferritin levels were within 50 ng/mL of the baseline value at first year follow-up. The “worsened ferritin group” (*n* = 53) was those subjects whose ferritin levels were more than 50 ng/mL higher than baseline value and the “improved ferritin group” (*n* = 26) was those subjects whose ferritin levels were more than 50 ng/mL lower than the baseline value at first year follow-up. The bivariate analysis of baseline demographic, clinical and laboratory factors at one-year and two-year follow-up for subjects with improved serum ferritin level versus those with worsened ferritin level is completed.

A separate analysis on three groups, as worsened ferritin, improved ferritin and unchanged ferritin at one-year follow-up was completed and is provided in the Supplementary Materials, Table S1.

### Statistical Methods

Continuous variables were described as means and standard deviations (SD) or, if the distribution of data was skewed, then as medians with upper and lower quartiles. Nominal variables were described as frequencies and percentages and differences between the variables were tested using the chi-squared test. The Wilcoxon signed-rank tests and paired *t*-tests were utilized to compare the outcomes of two matched pair groups. For more than two matched pair groups, a general linear mixed model was used with and without controlling for the confounder variables. The model was adjusted for primary etiology, age, BMI, the anatomical location of access (radial, brachial, femoral), the number of AVF submitted per study center (<5 cases, 5–10 cases, >10 cases) and HD vintage at AVF/AVG creation. A random intercept with an unstructured covariance matrix was used in all the models. Model adequacies were checked and satisfied. Statistical significance was set at *p* < 0.05 for all analysis. Statistical analyses were performed using the R statistical software (version 3.6.2; the R Foundation for Statistical Computing, Vienna, Austria).

## 3. Results

### 3.1. Subjects

The baseline demographic and clinical information of the 98 children receiving their first AVF/AVG has been previously described in detail [28,30]. A brief summary of these baseline characteristics is included in Table 1. There were 98 children who were converted from TCC to AVF/AVG during this study. AVF was created for 87 and AVG was created for 11 children. Both groups were comparable in age, demographic data, primary etiology of ESRD and race. The AVF group had less prior TCC use and shorter HD vintage prior to PVA creation when compared to AVG group, as seen in Table 1.

### 3.2. Serum Ferritin Levels

The mean serum ferritin level at PVA creation (TCC.Ferritin) was 562.64 ± 492.34 ng/mL. In the first year of AVF/AVG, the mean serum ferritin (PVA1.Ferritin) was 753.84 ± 561.54 ng/mL, which is statistically higher than the baseline level (*p* < 0.001). In the second year of PVA, the mean serum ferritin (PVA2.Ferritin) remained significantly higher than TCC.Ferritin (0.004) and statistically indifferent to PVA1.Ferritin (*p* = NS), as shown in Table 2. Serum Hct, albumin and single Kt/V all statistically improved at one-year follow-up and that improved level was maintained in the second year follow-up of PVA, as seen in Table 2.

Among these subjects, two ferritin trajectories were demonstrated over time: the majority demonstrating higher median serum ferritin levels at PVA1.Ferritin (worsened ferritin group) (*n* = 53) and a second group achieving lower serum ferritin (improved ferritin group) at PVA1.Ferritin (*n* = 26). Compared to worsened ferritin group, the improved ferritin group had significantly higher median serum ferritin levels at TCC.Ferritin (856 [403; 1320] vs. 305 [173; 458], respectively, *p* < 0.001), as shown in Table 3. At PVA1.Ferritin observation, the worsened ferritin group demonstrated statistically higher median serum ferritin level compared to the improved ferritin group (767 [535; 1013] vs. 467 [144; 750], respectively *p* = 0.002). At PVA2.Ferritin observation, the improved and worsened ferritin groups demonstrated statistically indifferent serum ferritin levels (678 [198; 832] vs. 762 [506; 1061], respectively *p* = 0.120).

These two groups were similar according to demographic characteristics, past TCC vintage and HD efficacy markers at one-year follow-up. The exception was serum albumin, which was statistically higher at PVA1.Albumin for the worsened ferritin group. Table 3.

A multiple linear regression model revealed three predictors for PVA1.Ferritin: steroid-resistant nephrotic syndrome (SRNS) as the primary etiology (*p* = 0.035), PVA reported from a HD center contributing >10 PVA to the study (*p* = 0.049) and baseline serum ferritin level at PVA creation (TCC.Ferritin) (*p* = 0.017). As predictors of PVA1.Ferritin, children with steroid-resistant nephrotic syndrome as their primary etiology demonstrated significantly lower serum ferritin levels (β = −438.93, 95% CI: [−845.41, −32.46], *p* = 0.035) compared to those with CAKUT, and children treated at a HD center that contributed >10 cases had a significantly higher level of serum ferritin (β = 319.85, 95% CI: [2.02, 637.68], *p* = 0.049) compared to those treated at centers that enrolled 5–10 cases. Furthermore, for every 50 mg/mL increase of serum ferritin at TCC.Ferritin stage, there was an associated significant increase for PVA1.Ferritin (β = 17.96, 95% CI: [3.38, 32.53], *p* = 0.017). Although not reaching statistically significant level, the femoral PVA compared to brachial PVA, AVF compared to AVG, and chronic glomerulonephritis compared to CAKUT demonstrated lower serum ferritin levels at PVA1.Ferritin Table 4.

### 3.3. Ferritin Trajectory over Time

**Figure 1:**
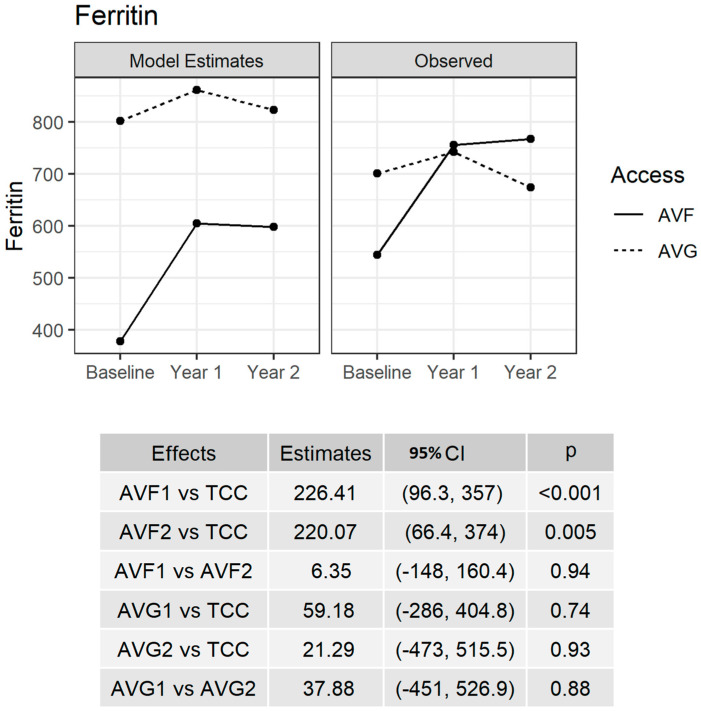
**Adjusted model for serum ferritin. Observed estimates (right-hand graph) and model estimates (left-hand graph and bottom table) for serum ferritin (ng/mL).** The model was adjusted for primary etiology, age, BMI, anatomical location of access (radial, brachial, femoral), the number of AVF submitted per study center (<5 cases, 5–10 cases, >10 cases) and HD vintage at PVA creation. Please note that TCC represents the Ferritin baseline value; AVF1 and AVF2 represent the year 1 follow-up Ferritin value and year 2 follow-up Ferritin value for the AVF group, respectively; and AVG1 and AVG2 represent the year 1 follow-up value and year 2 follow-up value of Ferritin for AVG group, respectively. CI: 95th percentile confidence interval, P: *p*-value.

Ferritin trajectories following conversion to AVF were further analyzed using adjusted models. AVF1.Ferritin was significantly increased in comparison to TCC.Ferritin (*p* < 0.001). AVF2.Ferritin remained significantly higher compared to TCC.Ferritin (*p* = 0.005). There was no difference between AVF1.Ferritin and AVF2.Ferritin (*p* = NS). The conversion to AVG was not associated with a significant difference in serum ferritin levels. AVG1.Ferritin and AVG2.Ferritin were indifferent compared to TCC.Ferritin (*p* = NS and *p* = NS, respectively), as shown in Figure 1.

### 3.4. Association of Serum Ferritin Levels to Serum Hct Levels at Three Data Points

**Figure 2:**
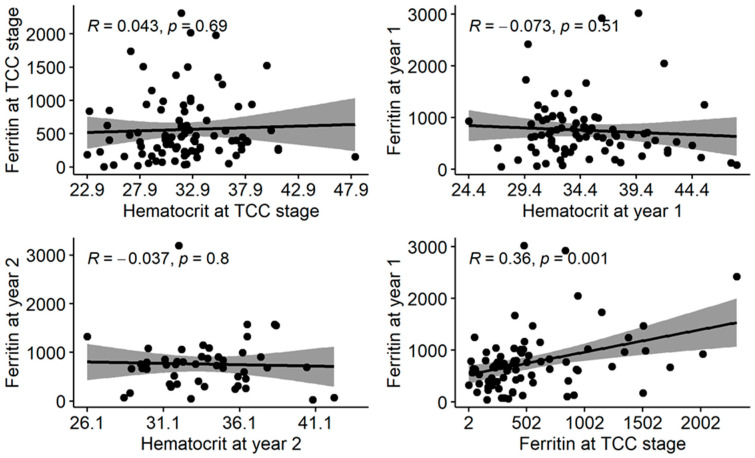
**Comparative graphs of ferritin vs. hematocrit at various data points during the two years of follow-up.** There was no statistically significant linear association between serum ferritin and hematocrit levels at all three data points. PVA1.Ferritin demonstrated a significant linear association with ferritin at PVA creation (right, bottom graph).

Ferritin levels were plotted against the Hct value at three data points to study their associations. There was no significant linear association between Ferritin and Hct at the time of PVA creation (TCC.Ferritin), at first year (PVA1.Ferritin) or second year of PVA (PVA2.Ferritin) (*p* = 0.69, *p* = 0.51, *p* = 0.8, respectively). PVA1.Ferritin showed a significant linear association with TCC.Ferritin (*p* = 0.001), as shown in Figure 2.

## 4. Discussion

In our study, using a contemporary cohort of 98 children on maintenance HD, serum ferritin levels were elevated according to KDOQI and KDIGO anemia guidelines while using TCC as vascular access, which was further increased during the first year after conversion to PVA and reached a plateau at the second year of follow-up. When the analysis was stratified according to the pattern of trajectory, about half of the subjects demonstrated worsened ferritin levels at one-year, a quarter of subjects displayed improved ferritin levels and the last quarter exhibited unchanged ferritin levels. There was no difference in clinical or baseline laboratory values between subjects with the worsened ferritin and the improved ferritin levels, except that TCC.Ferritin was statistically higher for the improved group. There were three predictors of PVA1.Ferritin: serum ferritin at baseline was able to predict PVA1.Ferritin, subjects with SRNS as their primary etiology demonstrated significantly lower PVA1.Ferritin compared to CAKUT patients and subjects from HD centers that treated >10 patients revealed significantly higher PVA1.Ferritin compared to centers that enrolled 5–10 subjects. 

One of the clinical uses of serum ferritin is to guide the clinician in regard to the use and the amount of parenteral iron replacement for the treatment of anemia for ESRD patients [13,15,16]. The registry reports from adult HD patients demonstrate that the average serum ferritin levels have been increasing in the USA in the past 20 years [31,32]. Compared to 2006 KDOQI guidelines, 2012 KDIGO anemia guidelines recommends higher cut-off values of serum transferrin saturation (<30%) and accepts higher serum ferritin levels (<500 ng/mL) for the initiation of parenteral iron treatments [6,19]. Furthermore, it has been demonstrated that parenteral iron treatments can further improve serum Hgb levels when serum transferrin saturation is <25%, even if serum ferritin levels are elevated >500 ng/mL [16,23,33]. As a result of all these publications, the use of parenteral iron has increased for HD patients [32,33,34]. The cost of ESA and the bundle of ESRD payments may have also favored parenteral iron utilization [27,31,32]. As a result, our observed increases in serum ferritin levels in maintenance HD patients may simply be result of the more liberal use of parenteral iron. Further clues that serum ferritin may be a modifiable factor that is strongly related to parenteral iron administration may be evident from our findings that if subjects revealed dangerously high levels of serum ferritin at PVA creation, their levels significantly improved during the first year of follow-up, perhaps due to restricted iron dosing. On the contrary, subjects with relatively lower serum ferritin levels, certainly within the KDIGO allowed cut-offs, displayed worsened levels, again perhaps due to clinicians being more liberal in iron dosing for these subjects. This suggestion may further be supported by the fact that there was no difference in baseline laboratory or clinical values between subjects of the two ferritin trajectory groups, except PVA1.Albumin.

Serum ferritin has also been presented as a marker of subtle ongoing sub-clinical inflammation in maintenance HD patients [17,22,23]. Malnutrition, cachexia, the use of TCC as vascular access as well as the HD treatment itself may be some of the contributors of this described “hemodialysis-associated inflammation” [22,23]. Since the use of TCC is demonstrated to be associated with elevated inflammatory markers in pediatric HD patients when compared to the use of PVA, one of our intentions in this study was to observe the change in serum ferritin as an inflammatory marker after conversion to PVA [20,21]. Two major deficiencies in this study in taking this approach was the lack of a control group that remained on TCC for the follow-up period and the lack of other inflammatory markers, such as C-reactive protein (CRP) or erythrocyte sedimentation rate (ESR), to trend over time along with serum ferritin levels. Even though there was no association between ferritin levels and serum Hct over two years, serum albumin, serum Hct and Kt/V improved during follow-up, which suggests that inflammation may not be the major driver of the trajectory of serum ferritin. It may be clear that the per se improvement of Kt/V and URR is secondary to better clearance due to a switch in vascular access. However, improved uremic milieu, demonstrated by higher Kt/V and higher URR, may contribute to improved hemodialysis-induced inflammation. Moreover, the significant improvement of serum albumin, being a negative inflammatory marker and improvement in serum hematocrit, may also suggest improved hemodialysis-induced inflammation. However, we fully recognize that, depending on future requirements, this question will need to be reconsidered in future studies with appropriate control group and checking common inflammatory markers overtime. 

There were three predictors of PVA1.Ferritin using a multiple linear regression model. The baseline serum ferritin predicting PVA1.Ferritin may suggest that this outcome is the result of standard of care in anemia management, which maintains similar impacts over time. SRNS subjects presenting with significantly lower serum ferritin compared to CAKUT subjects may be related to the standard treatment protocols of these patients, involving immunosuppressants (IS). Longtime exposure to IS prior to reaching ESRD may have some long-lasting effects. Alternatively, this outcome may be related to the erythropoietin secretion ability of the native failing kidneys between the two etiologies, which may suggest that structurally damaged kidneys may not be as efficient in this task compared to kidneys with primary glomerular disease [35]. This may contribute to a deeper anemia and may therefore stimulate more parenteral iron use [31,32]. Finally, it is very difficult to speculate why subjects from centers that enrolled >10 patients had higher serum ferritin levels.

There are certain reported concerns about the validity of serum ferritin levels to be utilized as the only marker for the body’s iron stores and as help to decide on parenteral iron dosing for hemodialysis patients [13,14,16]. At low levels of serum ferritin (<200 ng/mL), it stands as a valuable marker for iron deficiency [11,12]. That is, if the body iron stores are depleted, the serum ferritin will be low and this level will solely reflect the body iron stores [11]. However, once the body acquires adequate iron stores, serum ferritin claims its “acute-phase reactant” properties [16,22,23]. This was demonstrated by showing IL-1ß inducing ferritin gene expression through pathways that are different from iron-mediated ferritin expression [17,18]. Therefore, once serum ferritin is >200 ng/mL, it likely becomes a biomarker for both iron storage as well as the level of inflammation [14,22,23]. It was demonstrated in adult maintenance HD patients that moderately high serum ferritin (>500 ng/mL) is strongly associated with inflammatory markers, such as C-reactive protein (CRP) and IL-6 levels [22,23]. In fact, serum transferrin saturation and IL-6 can equally predict moderately elevated serum ferritin levels, while the combination of the two improves the prediction ability [23]. Lastly, serum ferritin levels associate strongly with the severity of malnutrition in HD patients [22]. The practical implications of these findings are that patients may be deprived of parenteral iron treatments because of the misinterpretation of elevated serum ferritin levels, when in reality, those levels may be driven by ongoing inflammation [13,16,17]. Secondly, due to its association with inflammation and malnutrition, serum ferritin levels may be utilized as a biomarker for increased morbidity and mortality and not recognizing them as such may harm our ability to provide the necessary care for our patients [26,29,35]. For the above-stated reasons, pediatric HD physicians should seek investigations trying to understand whether serum ferritin levels can provide similar insights for children on chronic HD as it does for adults [4,5].

The findings of our study have several limitations. Most relevant to our results, we did not collect the data points for parenteral iron dosing, ESA frequency and dosing and serum transferrin saturation levels while analyzing the changes in serum ferritin levels. The lack of a control group of children kept on TCC as a vascular access prohibit us from concluding that PVA was the reason for the noted trajectory in serum ferritin. We did not have data points for some common inflammatory markers in conjunction with changes in ferritin levels. Children enrolled in this study spent quite a long time undergoing HD (a mean time of almost three years), suggesting they had certain contraindications to kidney transplantation. Some of these contraindications could also have affected serum ferritin levels. Finally, contrary to the AVF group, the size of the AVG group was too small to perform reliable statistical calculations. During the calculation of the models for AVG outcomes, the lack of some data points did not allow the utilization of all 11 of the AVG subjects, which may have contributed to the conflicting findings compared to the observed values. However, the data have certain strengths, most notably that each subject presents as their own control. Since all subjects were switched from TCC to AVF/AVG during their chronic HD vintage, there are fewer patient-related factors impacting the differences between groups, which is a limitation for studies comparing two separate group of subjects utilizing PVA and TCC. In addition, the duration of follow-up gives enough time to demonstrate the long-term effects of the change in vascular access.

## 5. Conclusions

In conclusion, this retrospective multi-center study suggests that contemporary pediatric maintenance HD patients have elevated serum ferritin levels while utilizing TCC, which continues to increase for most of the subjects following conversion to AVF/AVG. This increase in serum ferritin levels is not associated with serum Hct levels and yet is accompanied with a significant improvement in other markers of HD efficacy. The serum ferritin level seems receptive to modifications by the standard of care of anemia management, possibly by avoiding or limiting the parenteral iron exposure. Systemic, sub-clinical HD associated inflammation is less likely to be the prominent reason for elevated serum ferritin as both serum albumin and single pool Kt/V improved during the follow-up. This study would be more informative if a matched-control group of subjects who were started on HD with TCC and was never converted to AVF/AVG with three years follow-up could be included. Considering the long years of ESRD care for the pediatric patients, this early elevated serum ferritin levels may contribute to adverse outcomes [8,9,10]. Therefore, it is important to design and execute the prospective trials studying the impact of anemia management strategies and inflammation on serum ferritin levels in pediatric maintenance HD patients and the effect of using TCC vs. AVF/AVG on serum ferritin levels [1,2,4]. More importantly, serum ferritin needs to be evaluated as a potential biomarker for pediatric ESRD patients, testing its ability to predict adverse outcomes for these children [24,25,26,29].

## Figures and Tables

**Table 1:** Demographics for the included subjects.

Baseline Demographic Information	All Subjects *n* = 98	AVG Subjects *n* = 11	AVF Subjects *n* = 87	*p*-Value
Average number of TCC used, mean ± sd	2.9 ± 1.1	3.2 ± 1.4	1.60 ± 1.37	**0.037**
Average duration of TCC vintage (months) mean ± sd	10.4 ± 17.3	28.8 ± 29.5	8.0 ± 13.6	**0.043**
CAKUT as primary etiology of ESRD	33 (33.7%)	3 (27.3%)	30 (34.5%)	0.746
Male (%)	58 (59.2%)	5 (45.5%)	53 (60.9%)	0.348
African American (%)	48 (49%)	8 (72.7%)	40 (46.0%)	0.176
Subjects < 10 years of age	9 (9.2%)	1 (9.1%)	8 (9.2%)	0.99
Age at AVF/AVG creation (years), median (IQR)	15.3 (13.2; 17.11)	15.2 (13.2; 15.5)	15.2 (13.2; 17.2)	0.468
Weight at AVF/AVG creation (kg), median (IQR)	48.7 (39.1; 64.8)	47.4 (43.0; 60.3)	48.9 (38.9; 64.5)	0.879
Height at AVF/AVG creation (m), median (IQR)	1.55 (1.48; 1.65)	1.52 (1.47; 1.61)	1.55 (1.48; 1.65)	0.57
Body mass index at AVF/AVG creation (BMI, kg/m^2^), median (IQR)	20.0 (17.2; 25.6)	19.1 (17.2; 26.6)	20.1 (17.3; 24.1)	0.973

Cells are represented as *n* (%) or mean ± SD (standard deviation) or median (IQR: 1st quartile; 3rd quartile). *p*-value refers to AVF group vs. AVG group. Statistically significant *p* values are reported in bold.

**Table 2:** Change in ferritin values after conversion to AVF/AVG over two years period in comparison to the change in other HD efficacy markers, Hct, albumin and Kt/V (*n* = 98).

HD Biomarkers	TCC (*n* = 98)	PVA.1	*p* Value	PVA.2	*p* Value *	*p* Value **
Ferritin (ng/mL) mean ± sd	562.64 ± 492.34	753.84 ± 561.54	**<0.001**	759.60 ± 528.11	**0.004**	0.77
Hematocrit (%), mean ± sd	32.04 ± 4.43	34.92 ± 4.66	**<0.0001**	34.04 ± 3.64	**0.003**	0.24
Albumin (gram/dL), mean ± sd	3.59 ± 0.76	3.91 ± 0.47	**<0.0001**	3.88 ± 0.53	**0.001**	0.91
Single pool Kt/V, mean ± sd	1.49 ± 0.45	1.67 ± 0.41	**0.02**	1.63 ± 0.26	**0.01**	0.45

TCC: The value of the HD efficacy marker at the time of PVA creation. PVA.1: the value of the HD efficacy marker at 1 year after PVA creation. PVA.2: the value of the HD efficacy marker at 2 years after PVA creation. *p* value, *p* value *, and *p* value ** are computed using paired *t*-tests for TCC vs. PVA.1, TCC vs. PVA.2 and PVA.1 vs. PVA.2, respectively. Statistically significant *p* values are reported in bold.

**Table 3:** Bivariate analysis of baseline demographic, clinical and laboratory factors at one-year and two-year follow-up for subjects with improved serum ferritin level versus those with worsened ferritin level (total *n* = 79).

Predictors	All Subjects (*n* = 79)	Improved Ferritin at PVA1 (*n* = 26)	Worsened Ferritin at PVA1 (*n* = 53)	*p*-Value Overall
TCC.Ferritin, median [IQR]	398 [230; 700]	856 [403; 1320]	305 [173; 458]	**<0.001**
PVA1.Ferritin, median [IQR]	675 [398; 964]	467 [144; 750]	767 [535; 1013]	**0.002**
PVA2.Ferritin, median [IQR]	746 [380; 910]	678 [198; 832]	762 [506; 1061]	**0.120**
Age at AVF/AVG creation (years), median [IQR]	15.3 [12.9; 17.1]	14.4 [11.2; 16.7]	15.5 [14.0; 17.2]	0.317
Weight at AVF/AVG creation (kg), median [IQR]	48.3 [36.4; 64.6]	43.2 [32.1; 54.6]	50.1 [39.5; 67.0]	0.11
Height at AVF/AVG creation (m), median [IQR]	1.55 [1.48; 1.65]	1.52 [1.42; 1.62]	1.57 [1.50; 1.65]	0.269
BMI at AVF/AVG creation (kg/m^2^), median [IQR]	19.7 [17.1; 26.3]	18.7 [16.6; 20.8]	20.4 [17.4; 27.5]	0.138
Male	48 (60.8%)	18 (69.2%)	30 (56.6%)	0.404
African American	38 (48.1%)	17 (65.4%)	21 (39.6%)	0.056
CAKUT as etiology	29 (36.7%)	7 (26.9%)	22 (41.5%)	0.310
Primary etiology #				0.113
Congenital	29 (36.7%)	7 (26.9%)	22 (41.5%)	
Glomerulonephritis	21 (26.6%)	7 (26.9%)	14 (26.4%)	
Other	16 (20.3%)	4 (15.4%)	12 (22.6%)	
SRNS	13 (16.5%)	8 (30.8%)	5 (9.43%)	
Average duration of TCC vintage prior to PVA (months), median [IQR]	10.5 ± 17.4	11.3 ± 14.7	10.27 ± 13.2	0.101
PVA1.Hct	34.0 [31.7; 37.4]	33.4 [30.8; 36.9]	34.3 [31.9; 37.5]	0.425
PVA1.Albumin	**3.90 [3.70; 4.20]**	**3.80 [3.60; 4.00]**	**4.00 [3.70; 4.30]**	**0.021**
PVA1.Kt/V	1.65 [1.39; 1.90]	1.71 [1.42; 2.03]	1.65 [1.39; 1.87]	0.581
Conversion to PVA				0.722
AVG (*n* = 10)	10 (12.7%)	4 (15.4%)	6 (11.3%)	
AVF (*n*= 69)	69 (87.3%)	22 (84.6%)	47 (88.7%)	

Cells are presented as *n* (%) or median (IQR: 1st quartile; 3rd quartile). Differences in the continuous data were tested using the Mann–Whitney U-test and differences in the categorical data were tested using the chi-squared test. #: Primary etiology was evaluated in four groups. Statistically significant *p* values are reported in bold.

**Table 4:** Association between PVA1.Ferritin and the associated predictors using a multiple linear regression model. Statistically significant *p* values are reported in bold.

Predictors	ß (SE)	95% CI	*p*-Value
Baseline ferritin (per 50 ng/mL)	17.96 (7.30)	3.38–32.53	**0.017**
Age at AVF/AVG creation	1.49 (2.07)	5.62–2.65	0.475
Dialysis vintage at AVF/AVG creation	2.82 (5.21)	−7.58–13.23	0.590
BMI at AVF/AVG creation	1.72 (9.65)	−17.54–20.98	0.859
Primary etiology			
CAKUT (Reference)			
Chronic glomerulonephritis	−210.4 (165.62)	−541.06–120.27	0.208
SRNS	−438.93 (203.59)	−845.41–−32.46	**0.035**
Other	−156.6 (187.61)	−531.19–217.98	0.407
Study sites according to the PVA created			
5–10 AVF/AVG (Reference)			
<5 AVF/AVG	−102.98 (184.07)	−470.38–264.42	0.578
>10 AVF/AVG	319.85 (159.19)	2.02–637.68	**0.049**
AVF vs. AVG	−134.19 (247.88)	−629–360.72	0.590
AVF/AVG site			
Brachial AVF/AVG (Reference)			
Radial AVF/AVG	8.09 (138.79)	−269.02–285.19	0.954
Femoral AVF/AVG	−390 (344.04)	−1076–296.90	0.261

## Data Availability

All data generated or analysed during this study are included in this article. Further enquiries can be directed to the corresponding author.

## Supplementary Materials

The following supporting information can be downloaded at: https://www.mdpi.com/article/10.3390/jcm12134251/s1, Table S1: Table 3 Supplement, Bivariate analysis of baseline demographic, clinical and laboratory factors at one-year and two-year follow-up for subjects with improved serum ferritin level versus unchanged ferritin level versus those with worsened ferritin level according to one-year follow up ferritin results. (Total *n* = 79).

## Abbreviations

TCC.Ferritin	Serum ferritin level at the time of PVA creation.
PVA1.Ferritin	Serum ferritin level at first year of PVA.
PVA2.Ferritin	Serum ferritin level at second year of PVA.
AVF1.Ferritin	Serum ferritin level at first year of AVF.
AVF2.Ferritin	Serum ferritin level at second year of AVF.
AVG1.Ferritin	Serum ferritin level at first year of AVG.
AVG2.Ferritin	Serum ferritin level at second year of AVG.
