# Supplementary material for: Persistent Increase in Serum Ferritin Levels despite Converting to Permanent Vascular Access in Pediatric Hemodialysis Patients: Pediatric Nephrology Research Consortium Study
Source: J Clin Med. 2023 Jun 25;12(13):4251. doi: 10.3390/jcm12134251 (PMC10342380; doi:10.3390/jcm12134251)
Supplement: Supplementary file 1 [file jcm-12-04251-s001.zip › jcm-2447134-supplementary.pdf]

Table S1: Description of the groups:

Serum ferritin trajectories were evaluated in three groups according to the change in ferritin levels. The "*unchanged ferritin group*" included those subjects whose ferritin level was within 50 ng/ml of the baseline value at first year follow-up. The "*worsened ferritin group*" was those subjects whose ferritin level was more than 50 ng/ml higher than baseline value and the "*improved ferritin group*" was those subjects whose ferritin level was more than 50 ng/ml lower than baseline value at first year follow-up.

Table S1. Bivariate analysis of baseline demographic, clinical and laboratory factors at one-year and two-year follow-up for subjects with improved serum ferritin level versus unchanged ferritin level versus those with worsened ferritin level according to one-year follow up ferritin results. (Total N=79).

**Predictors**	**All Subjects (N=79)**	**Improved Ferritin at PVA1 (N=22)**	**Unchanged Ferritin at PVA1 (N=6)**	**Worsened Ferritin at PVA1 (N=51)**	***P* value overall**
TCC.Ferritin, median [IQR]	398 [230; 700]	856 [403; 1320]	496 [417; 909]	301 [170; 465]	**<0.001**
PVA1.Ferritin, median [IQR]	675 [398; 964]	392 [130; 681]	494 [440; 898]	769 [584; 1014]	**0.002**
PVA2.Ferritin, median [IQR]	746 [380; 910]	666 [230; 822]	935 [615; 1126]	761 [481; 954]	**0.252**
Age at AVF/AVG creation (years), median [IQR]	15.3 [12.9; 17.1]	14.4 [11.2; 16.7]	16.6 [14.8; 17.5]	15.4 [14.0; 17.0]	0.376

Weight at AVF/AVG creation (kg), median [IQR]	48.3 [36.4; 64.6]	43.0 [32.1; 54.6]	49.1 [44.3; 62.3]	50.1 [38.2; 65.6]	0.345
Height at AVF/AVG creation (m), median [IQR]	1.55 [1.48; 1.65]	1.51 [1.42; 1.56]	1.65 [1.57; 1.67]	1.57 [1.49; 1.65]	0.147
BMI at AVF/AVG creation (kg/m2), median [IQR]	19.7 [17.1; 26.3]	18.8 [17.1; 20.8]	19.0 [16.6; 23.0]	20.4 [17.2; 27.1]	0.607
Male	48 (60.8%)	14 (63.6%)	5 (83.3%)	29 (56.9%)	0.502
African American	38 (48.1%)	14 (63.6%)	4 (66.7%)	20 (39.2%)	0.095
CAKUT as etiology	29 (36.7%)	5 (22.7%)	2 (33.3%)	22 (43.1%)	0.255
Primary etiology#					0.229
Average duration of TCC vintage prior to PVA (months), median [IQR]	10.5 ± 17.4	11.2± 13.6	10.41± 12.2	10.32± 14.6	0.103
TCC.Hct	32.1 [29.2; 34.5]	31.5 (28.1;34.3)	32.9 [31.3; 35.2]	32.1 (30.0;33.6)	0.669
PVA1.Hct	34.0 [31.7; 37.4]	33.4 [30.5; 36.3]	34.5 [31.9; 39.2]	34.3 [32.0; 37.5]	0.611
PVA1.Albumin	3.90 [3.70; 4.20]	3.75 [3.60; 4.00]	4.00 [3.82; 4.10]	4.00 [3.70; 4.30]	0.089

PVA1.Kt/V	1.65 [1.39; 1.90]	1.79 [1.35; 2.08]	1.58 [1.37; 1.69]	1.65 [1.41; 1.89]	0.572
Conversion to PVA type					0.547
AVG (N=10)	10 (12.7%)	4 (18.2%)	0 (0.00%)	6 (11.8%)	
AVF (N= 69)	69 (87.3%)	18 (81.8%)	6 (100%)	45 (88.2%)	

Cells are presented as n (%) or median (1st quartile; 3rd quartile). Differences in the continuous data were tested using the Mann-Whitney U test and differences in the categorical data were tested using the Chi-squared test. #Primary etiology is evaluated in four groups. #Primary etiology groups were CAKUT, chronic glomerulonephritis, Steroid-resistant nephrotic syndrome (SRNS) and other. Hct.TCC: Hematocrit level at PVA creation.
